# Culturally-Tailored Education Programs to Address Health Literacy Deficits and Pervasive Health Disparities among Hispanics in Rural Shelbyville, Kentucky

**DOI:** 10.4172/2161-0711.1000250

**Published:** 2013-11-16

**Authors:** Irma N Ramos, Kenneth S Ramos, Aisa Boerner, Qiang He, Marco A Tavera-Garcia

**Affiliations:** 1 Departments of Environmental and Occupational Health Sciences, School of Public Health and Information Sciences, University of Louisville, USA; 2 Biochemistry and Molecular Biology, School of Medicine, University of Louisville, USA; 3 Shelby Prevention Program, Shelbyville, KY, USA; 4 Center for Environmental Genomics and Integrative Biology, University of Louisville, USA

**Keywords:** Culturally-sensitive health literacy programs, Health disparities, Hispanic health, Immigrant health

## Abstract

**Objectives:**

This investigation was conducted to evaluate the impact of culturally-tailored education on health knowledge among Hispanic residents of rural, Shelbyville, KY.

**Design:**

The program identified specific pathways to address health literacy deficits and disparities identified through a community-wide health assessment completed in 2010.

**Results:**

A total of 43 Hispanic males who shared deficiencies in community-wide health infrastructure were enrolled in the program. The curriculum included an introductory session followed by five, subject-specific, sessions offered on a weekly basis from February to April 2011. Pre/post-test assessments showed marked improvement in knowledge base for all participants after each session, most notably related to cardiovascular disease, diabetes and metabolic syndrome. The group reconvened in January 2012 for follow-up instruction on cardiovascular disease and diabetes, as well as global assessment of knowledge retention over a nine-month period.

Comparisons of pre/post testing in cardiovascular disease and diabetes, as well as global health-related knowledge showed significant gains for all parameters.

**Conclusions:**

Health education programs that embrace perceptions of the community of their own health, and that integrate knowledge into culturally-sensitive education, significantly improved health knowledge among Hispanic residents in rural Kentucky. Such gains may translate into sustainable improvements in health literacy and help reduce health disparities.

## Introduction

A significant challenge faced by Americans in the 21^st^ Century is their inability to understand and act on health information in ways that promote individual and community health. These deficiencies point toward the need for increased health literacy as individual patients and populations continue to be deficient in their ability to access, understand and use information to make informed health decisions. A major complicating factor is that health-related information is often disseminated through reading materials written at the high school level when most adults comprehend at the 7^th^ or 8^th^ grade level [1]. According to the first National Assessment of Health Literacy completed in 2003, only 12% of the US adult population has proficient health literacy, while over a third of US adults, that is, approximately 77 million people have basic or below basic health literacy [2].

The relationship between health literacy and health outcomes has been studied by several groups [3-6]. The US Department of Health and Human Services defines health literacy as the degree to which individuals have the capacity to obtain, process, and understand basic health information and services needed to make appropriate health decision [7]. Several studies have shown that individuals with limited health literacy have less knowledge of their own health status, lower self-management skills, and higher rates of chronic illnesses, poorer health status, and limited involvement in preventive care. Limited health literacy affects adults in all racial and ethnic groups, with the proportion of adults with basic or below basic health literacy ranging from 28% of White adults to 65% of Hispanics adults [2,8]. Disadvantaged immigrant populations are likely more vulnerable to the challenges posed by low health literacy given the inherent limitations posed by socioeconomic determinants, restricted access to health care, and linguistic challenges. The U.S. Department of Health and Human Services reports that linguistic differences among patients directly impact their health literacy levels, which in turn, contribute to increased prevalence of health disparities among elderly adults, racial/ethnic minorities, recent refugees and immigrants, low-income individuals, and non-native speakers of English [9]. And while promoting health literacy is a strategy to reduce disparities, it also improves the provision of patient-centered care [10].

To address these challenges in rural communities in Kentucky, particularly as it relates to Hispanic health, a community outreach and engagement program was established in Shelbyville, KY. Shelbyville is one of the most diverse rural communities in Kentucky and is located in close proximity to the Louisville metropolitan area. A recent cross-sectional study of the Hispanic population in Shelbyville revealed that the majority of those surveyed originated from Mexico, with the remaining population originating from Guatemala and Honduras [11]. Significant deficits in the ability of participants to manage their health and prevent disease, health knowledge and access to health care systems were identified. The primary health concerns identified included alcoholism, cancer, child health, diabetes, environment, nutrition, sexually transmitted diseases, and women’s health.

The theoretical framework of the present investigation is rooted on a train-the-trainer model of community education designed to overcome gaps in health knowledge that contribute to below-basic health literacy primarily in terms of self-care and access and utilization of health care services. The present study summarizes the results of a culturally-tailored health education program in Shelbyville, KY established to promote health literacy among underserved Hispanic residents. A one-group, pre/post test design implemented in two phases spaced nine months apart was employed to evaluate the pre-existing knowledge base of participants, knowledge acquisition and retention and impact of follow-up training on testing performance. The data show that implementation of this program was associated with improved pre/post testing performance both short-term, as well as nine months after completion of the intervention. As such, culturally-tailored educational intervention offered in sequential phases may be a useful strategy for sustained engagement and health knowledge retention among disadvantaged Hispanic populations.

## Materials and Methods

### Partnerships

Community partners included El Centro Latino and the Shelbyville Extension Services, along with several faith-based organizations, most notably Church of the Annunciation which made its facilities available for group meetings and assisted with recruitment efforts.

### Education

A curriculum entitled “ *The Environment and Your Health*” was developed with input from partnering organizations and pilot tested during completion of a comprehensive health and social assessment of the Hispanic community in Shelbyville [11]. This curriculum was tailored to address the specific needs and desires of Hispanic residents using data gathered through the community health assessment and feedback from community leaders. The syllabi for individual sessions are available upon request. The curriculum was structured in two phases offered nine months apart in order to evaluate knowledge retention and impact of follow-up training. Phase I consisted of six individual modules, including an introductory meeting and five, one hour sessions offered between February and April, 2011. All presentations were in Spanish, the language of preference of participants, and offered in an informal setting using audiovisual technology. Phase II started in January, 2012 and included repeat instruction in two of the five modules.

Specific modules in Phase I were developed to include general concepts and take-home messages of health prevention and promotion in the areas of *cardiovascular disease, nutrition, diabetes, metabolic syndrome* and *sexually-transmitted diseases.* This phase was carried out using a conventional pre/post educational approach. All instruction was provided at the middle school level to ensure adequate dissemination of information among participants. The cardiovascular module included discussions on obesity, dietary fat intake, heart attacks, smoking, alcohol, physical inactivity, hypertension and stroke. The diabetes module included discussions of the different types of diabetes, the role of insulin in diabetes, signs and symptoms of diabetes and diabetic dietary guidelines. The module on nutrition included a discussion of nutrients and caloric intake, body mass index, dietary recommendations for health living, healthy and health food choices. The module on metabolic syndrome was developed to extend previous discussions on cardiovascular disease, diabetes and nutrition, and to provide a more global perspective on the health risks associated with combined cardiovascular and metabolic pathologies. This module included discussions on the definition of metabolic syndrome, incidence and risk factors for disease occurrence. Lastly, the module on sexually-transmitted diseases was focused on HIV/AIDS and included discussions on viral transmission, impact of HIV on the Hispanic community and modes of prevention. Health education sessions were held within the community at El Centro Latino or Church of the Annunciation. Each session was designed to allow for easy updates and customization based on the feedback provided by participants.

Phase II involved follow-up health education on *cardiovascular disease and diabetes* at the request of program participants. This phase was designed to measure long term knowledge retention as evidenced by pre-testing performance, as well as the impact of prior educational intervention on post-testing performance and global knowledge retention.

### Testing

Pre- and post-testing performance was examined to evaluate baseline knowledge and short-term knowledge gains after each module. Formative evaluation was provided during the pre-assessment phase, followed by informal lectures and group discussion and summative evaluation at the end. A pre/post testing strategy was also employed during the *second phase* of health education, except that this phase was primarily designed to establish comparative measures of knowledge retention as a function of time (nine months in this case) and to evaluate the impact of the educational intervention on pre/post testing performance.

### Statistical analysis

**Figure 1:**
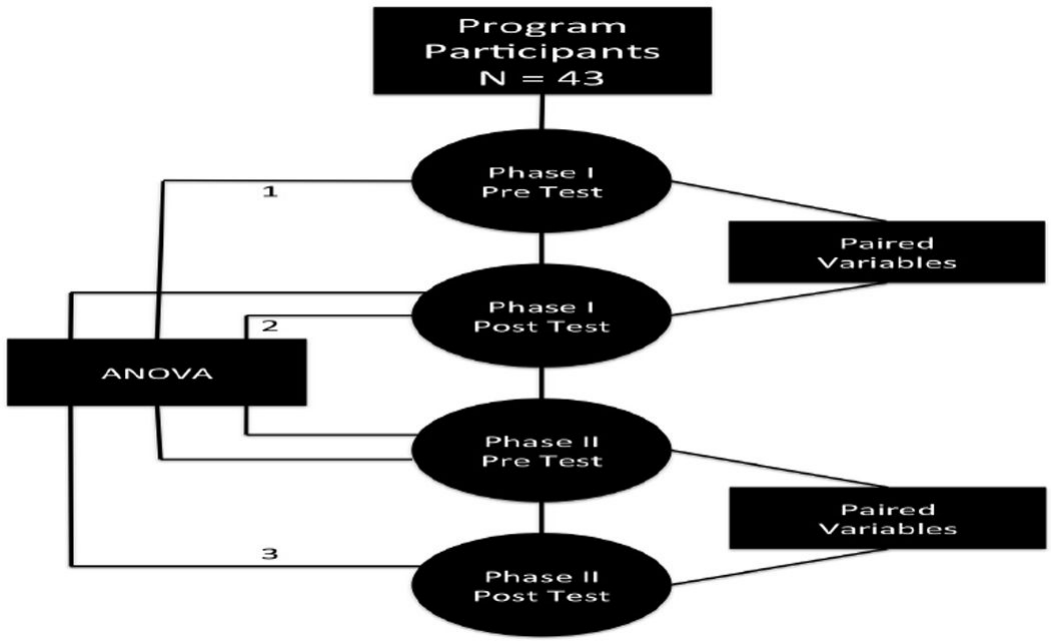
Statistical Analysis Scheme. 1=Baseline knowledge; 2=Knowledge retention over a 9 months period; and 3=Impact of the intervention.

Figure 1 shows a schematic of the statistical design. Pre- and post-test assessments were completed for all educational sessions and compared using paired t-tests, with pre and post-tests used as the paired variables. This approach was necessary given that it cannot be assumed that the two sets of data are independent, and in fact natural pairing of the data may exist. A level <.01 was accepted as significant. ANOVA and Fishers post hoc testing were employed to compare pre/post testing performance during phases I and II of the investigation. A level of <.01 was accepted as significant.

## Results

### Participants

Forty-three Hispanic males residing in Shelbyville, KY who regularly gathered at El Centro Latino to seek food assistance and/or temporary job placement during winter months were enrolled in the educational program. Given the periodic nature of the encounters, data was not available on length of stay in the Shelbyville area or level of education. Informal reports indicated that most participants were employed in the agricultural or horse farming industries during the summer and fall, leaving them to seek temporary employment during the winter months.

### Education

The educational program was implemented in two phases. The first phase included teaching of five modules on cardiovascular diseases, nutrition, diabetes, metabolic syndrome and sexually transmitted diseases. The program was initiated in February, 2011 and completed in April, 2011 and consisted of two-hour sessions inclusive of time for informal discussions at the beginning and end of each session, topical discussion for 1 hour and time for questions and answers as desired by program participants. Maximal attendance to the first phase of the program was 26 participants. The *second phase* was implemented in January, 2012 and involved repeat instruction on cardiovascular diseases and diabetes, as well as global assessment of knowledge retention. Maximal attendance to the second phase of the program was 35 participants.

**Figure 2:**
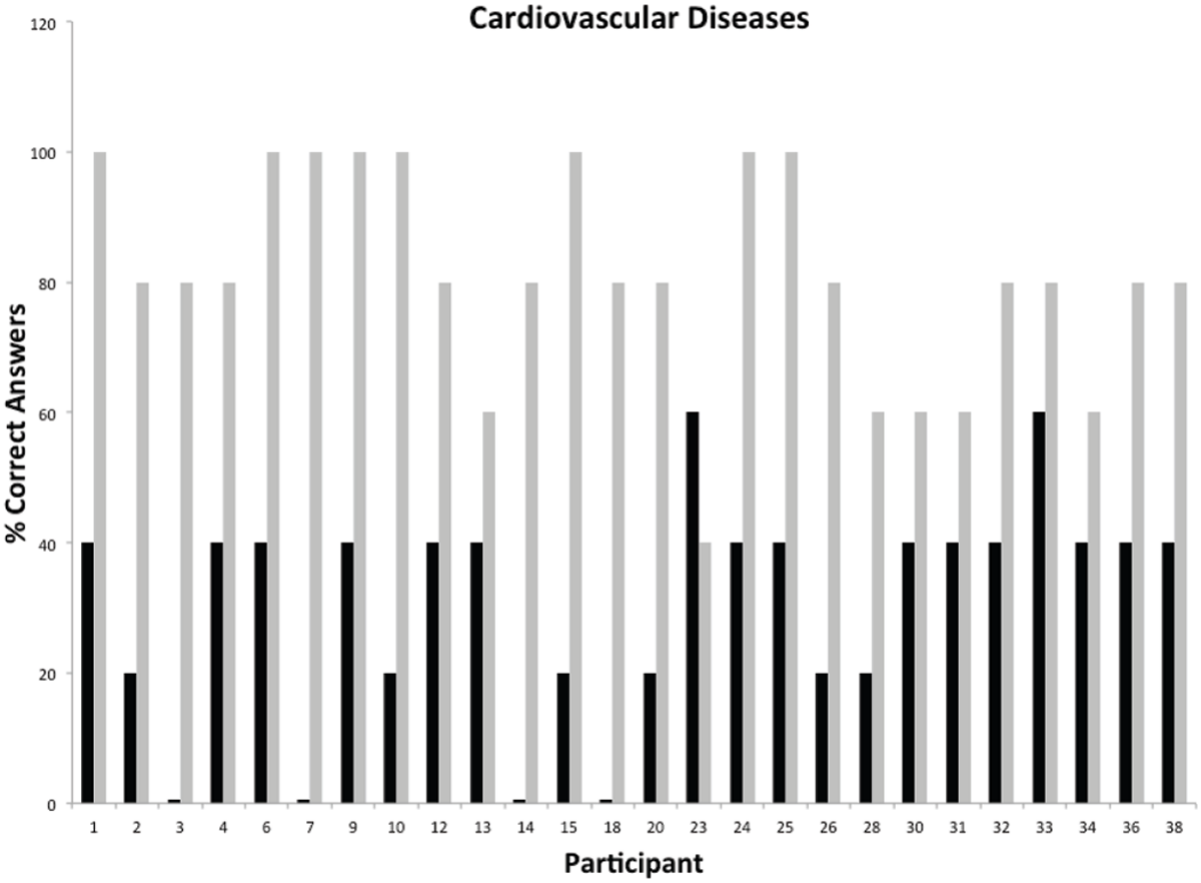
Short-Term Knowledge Gains in Cardiovascular Diseases by Individual Hispanic Participants Following Educational Intervention.

**Figure 3:**
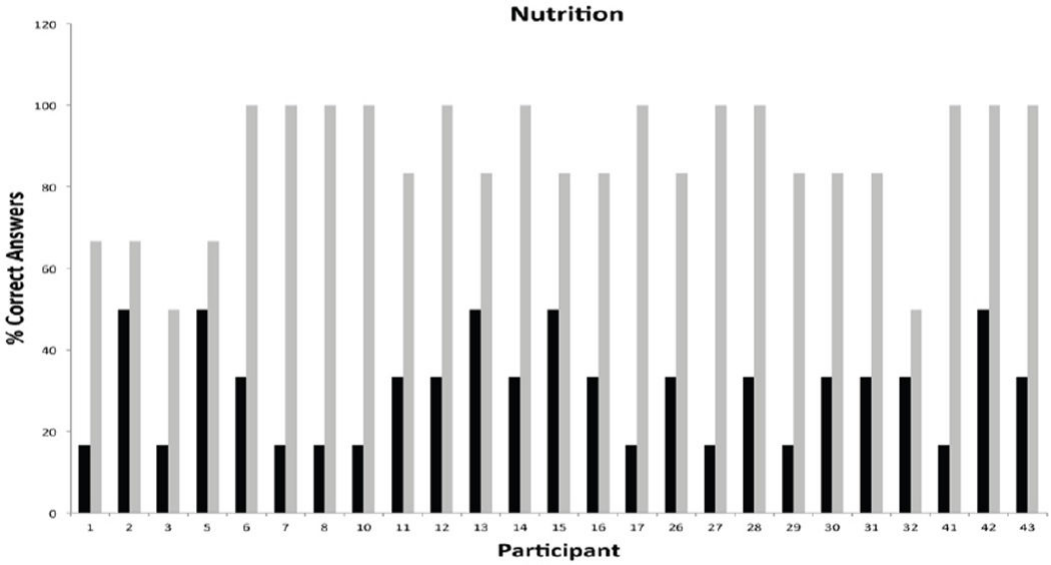
Short-Term Knowledge Gains in Diabetes by Individual Hispanic Participants Following Educational Intervention.

**Figure 4:**
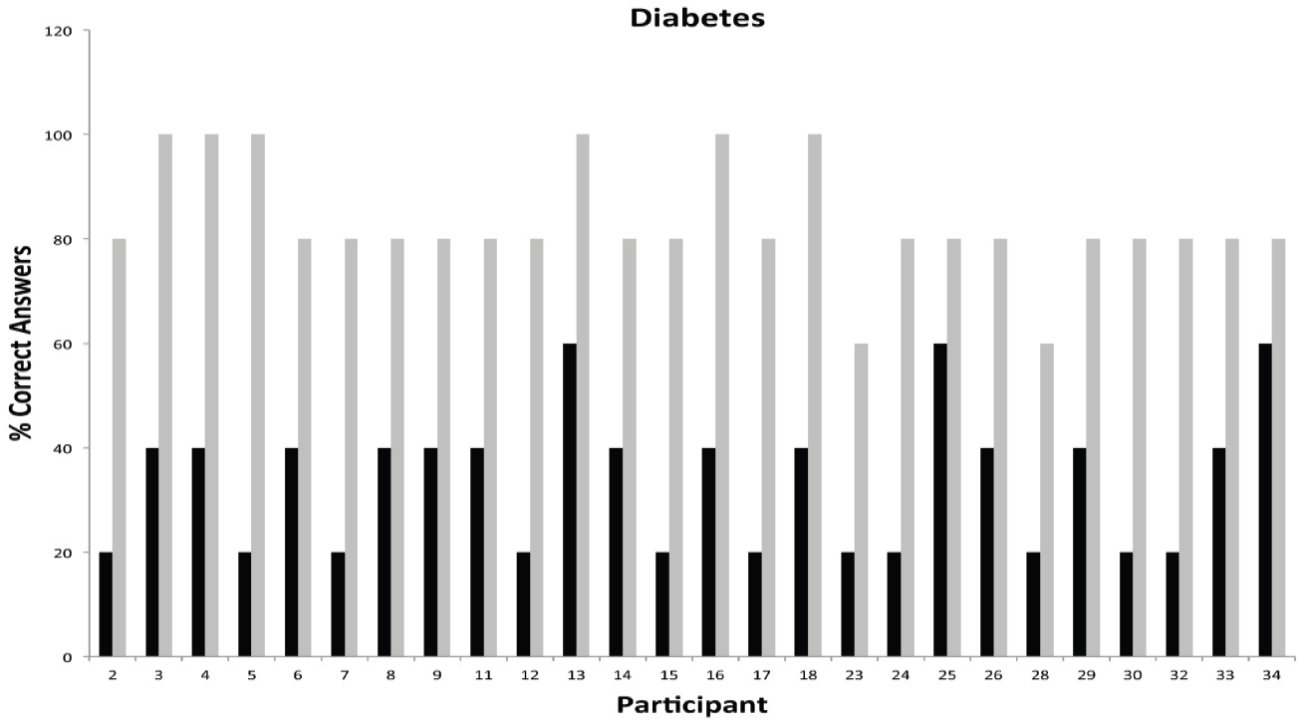
Short-Term Knowledge Gains in Diabetes by Individual Hispanic Participants Following Educational Intervention.

**Figure 5:**
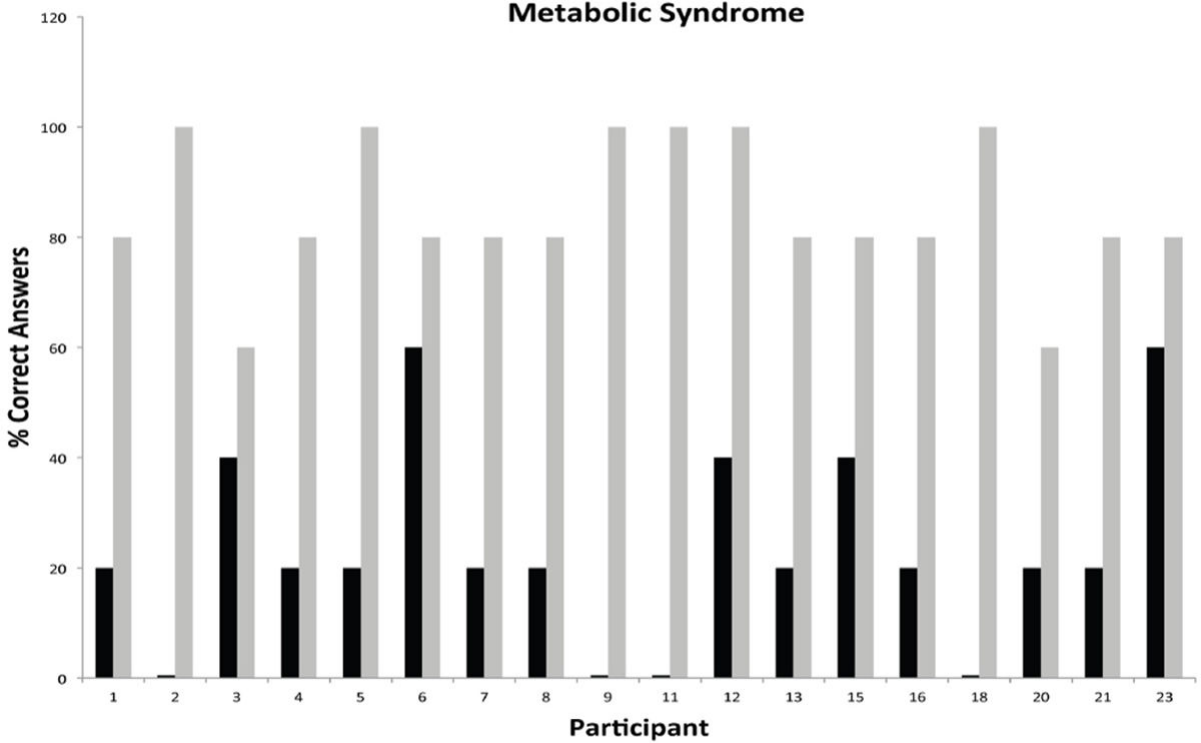
Short-Term Knowledge Gains in Metabolic Syndrome by Individual Hispanic Participants Following Educational Intervention.

**Figure 6:**
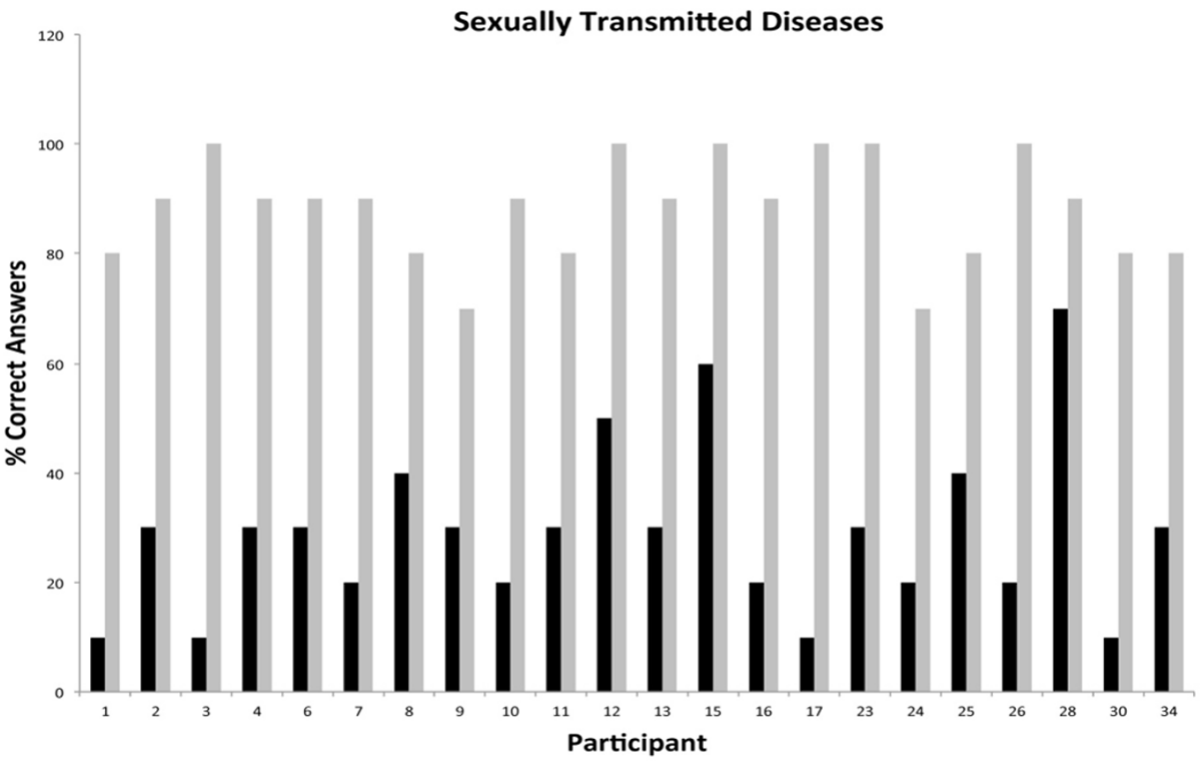
Short-Term Knowledge Gains in Sexually Transmitted Diseases by Individual Hispanic Participants Following Educational Intervention.

Participation during the phase I was consistently high for all modules and ranged from 18 to 26 participants. Each subject was assigned a unique identifier that was carried throughout the duration of the program for tracking of participation and performance. Unique identifiers were used to depict all graphical representations of the data in order to facilitate comparisons across individual modules and phases of the program. A measure of the pre-existing knowledge base and post-intervention performance for individual participants attending the cardiovascular module is presented in Figure 2. The data showed that participants had a modest knowledge base in this topical area, with only 4/26 participants missing all questions versus 16/26 participants scoring 40% or greater in the pretest. The greatest gap in knowledge prior to intervention was related to hidden sources of cholesterol in the diet and signs and symptoms of heart disease or stroke. Post-testing results showed remarkable improvements in overall knowledge among participants with 20/38 participants scoring 80% or better. The most significant knowledge gains were realized in the areas of cholesterol synthesis and storage in the human body, and high cholesterol as a known risk factor of heart attacks and stroke. The second module focused on generalized principles of nutrition. This module was attended by 25 participants with a pre-intervention knowledge base ranging from 17 to 50% (Figure 3). Participants were generally knowledgeable about the concept of nutrients, but unfamiliar with concepts of nutrition requirements, calorie counting, nutritional pyramid and body mass index. The overall knowledge base on this topic was weak with 9/25 participants scoring 17% and 20/25 scoring below 50%. As in the first module, post-testing performance showed remarkable short-term knowledge gains, with 20/25 participants scoring greater than 80% and 12/25 scoring 100%. Figure 4 shows the pre/post-testing performance of 26 participants attending the module on diabetes. Pre-intervention performance of participants ranged from 20 to 60%, with most participants scoring in the 20-40% range. Post testing showed marked improvements with 24/26 scoring 80% or better. The fourth module focused on metabolic syndrome, a topic which proved to be one of the most challenging because of significant deficits in pre-intervention knowledge base, with 4/18 participants missing all questions during pre-testing and 9/18 scoring at 20% (Figure 5). Significant knowledge gains were realized upon completion of the module with 16/18 participants scoring greater than 80% in the post-test. The last module focused on education on sexually transmitted diseases (Figure 6). While pre-testing performance was generally deficient with 17/22 participants scoring at or below 30%, it was interesting to find that all participants were familiar with the human immunodeficiency virus (HIV), but overwhelmingly unfamiliar with general modes of HIV transmission, needle sharing, heterosexual transmission and incidence of HIV among Hispanics. Significant improvements in knowledge base were measured during post-testing with 20/22 participants scoring at or greater than 80%.

**Figure 7:**
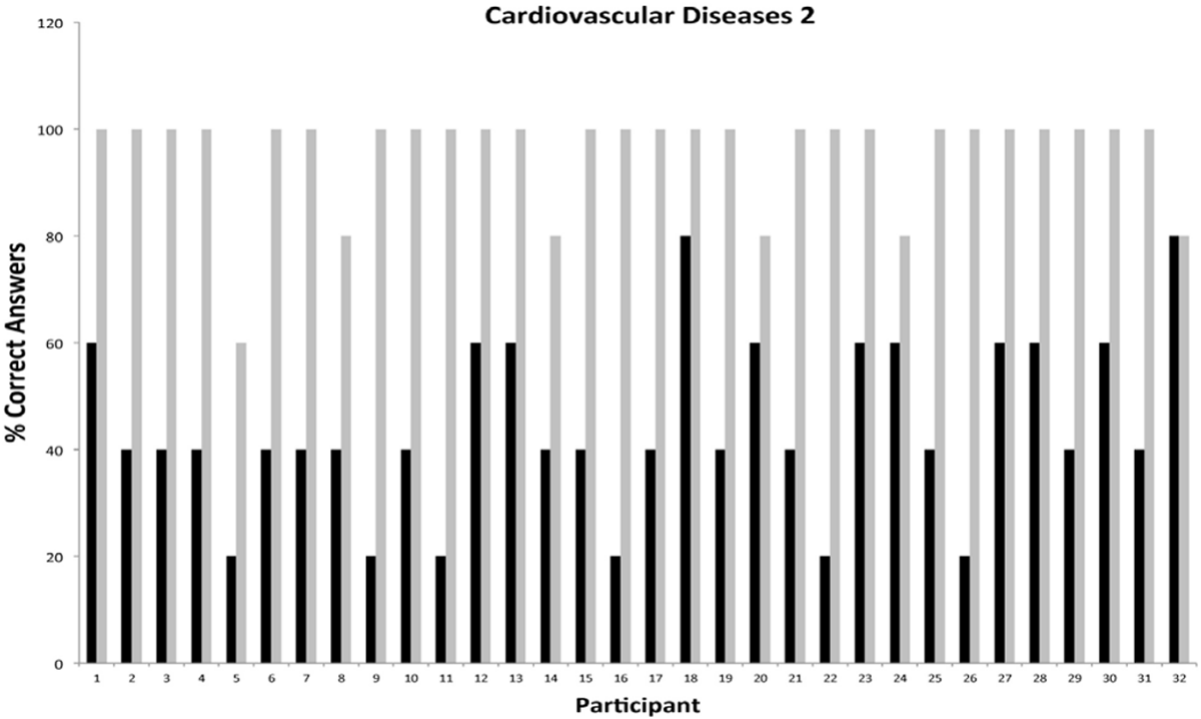
Knowledge Base and Reinforcement in Cardiovascular Diseases Nine Months Following Initial Educational Intervention Among Individual Hispanic Participants.

**Figure 8:**
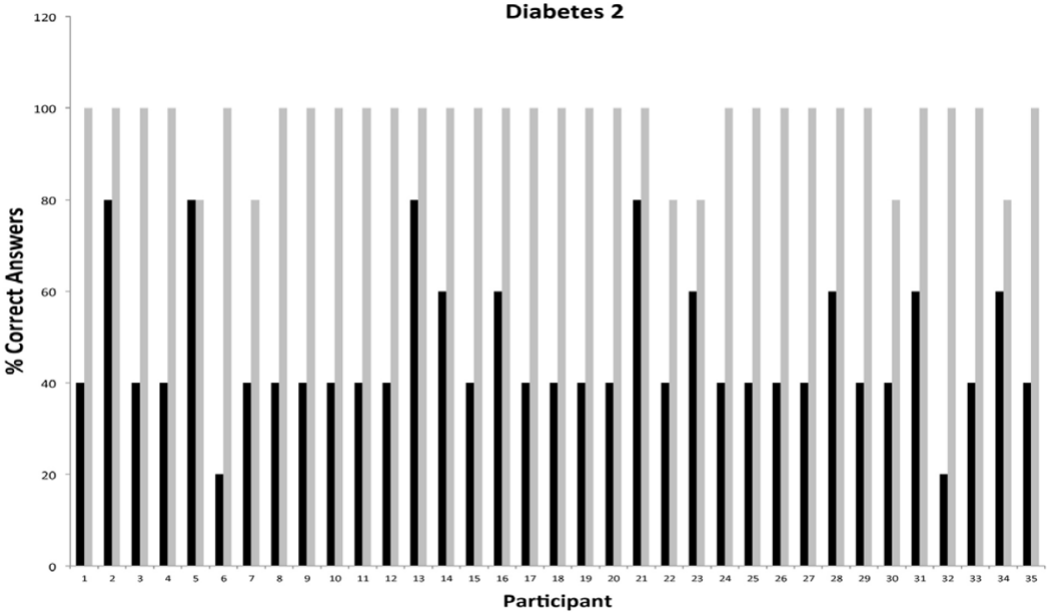
Knowledge Base and Reinforcement in Diabetes Nine Months Following Initial Educational Intervention Among Individual Hispanic Participants.

**Figure 9:**
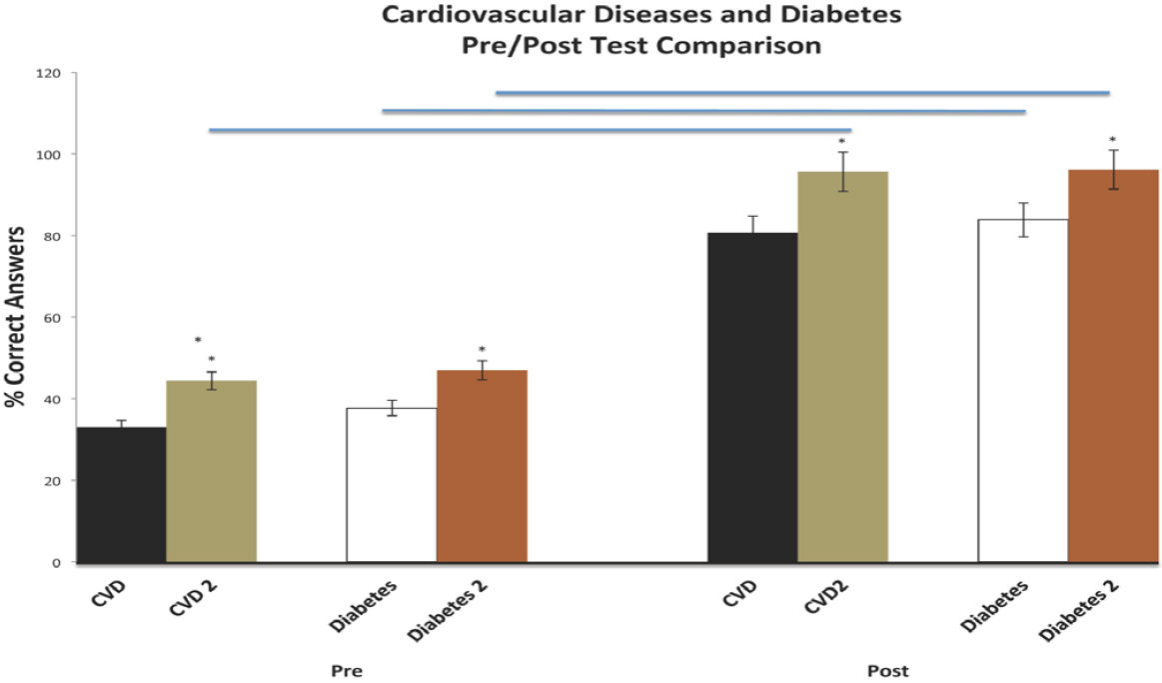
Pre-Post Testing Comparisons in the Areas of Cardiovascular Disease and Diabetes Over a Nine Month Interval Period of Educational Intervention. Asterisks denote significant differences in performance between first and second intervention, while bars denote significant differences between pre- and post-testing performance.

The second phase of the program focused strictly on cardiovascular diseases and diabetes education. This phase was offered nine months after completion of the *first phase* to evaluate and compare short- and longer-term knowledge gain. The results of pre-/post-testing performance in the areas of cardiovascular diseases and diabetes during phase II of the intervention are presented in Figures 7 and 8, respectively. Of note, 23 of 32 participants who completed the cardiovascular module during the first phase of the program enrolled in the second phase for an impressive reenrollment rate of 88%. Pre-test performance of participants in cardiovascular disease was consistently high with 26/32 scoring at or above 40%. Importantly, 16/23 participants showed improved pre-testing performance when compared to the first phase of the program; evidence of significant knowledge retention over a 9 month period after completion of education. In keeping with this interpretation, post-testing performance in cardiovascular was consistently improved compared to phase I with 31/32 participants scoring 80% or better, and 26/32 scoring 100%. For the diabetes module, 24/32 participants reenrolled in the program. Marked improvements in pre-testing performance were realized by participants, with 33/35 participants scoring 40% or better, and 10/32 scoring at 60% or better. Among return participants, 14/26 showed improved pre-testing performance, while 25/26 (96%) showed sustained pre-testing performance. Direct comparisons of cumulative pre-/post-test performance in cardiovascular diseases and diabetes showed significant improvements in both pre- and post-testing performance for participants who had re-enrolled in the program (Figure 9). Statistical analyses of the totality of the data were completed for pre-test and post-test as paired variables. All paired t-tests were significant (<.0001), indicating that educational intervention significantly affected their capacity to give correct answers.

**Figure 10:**
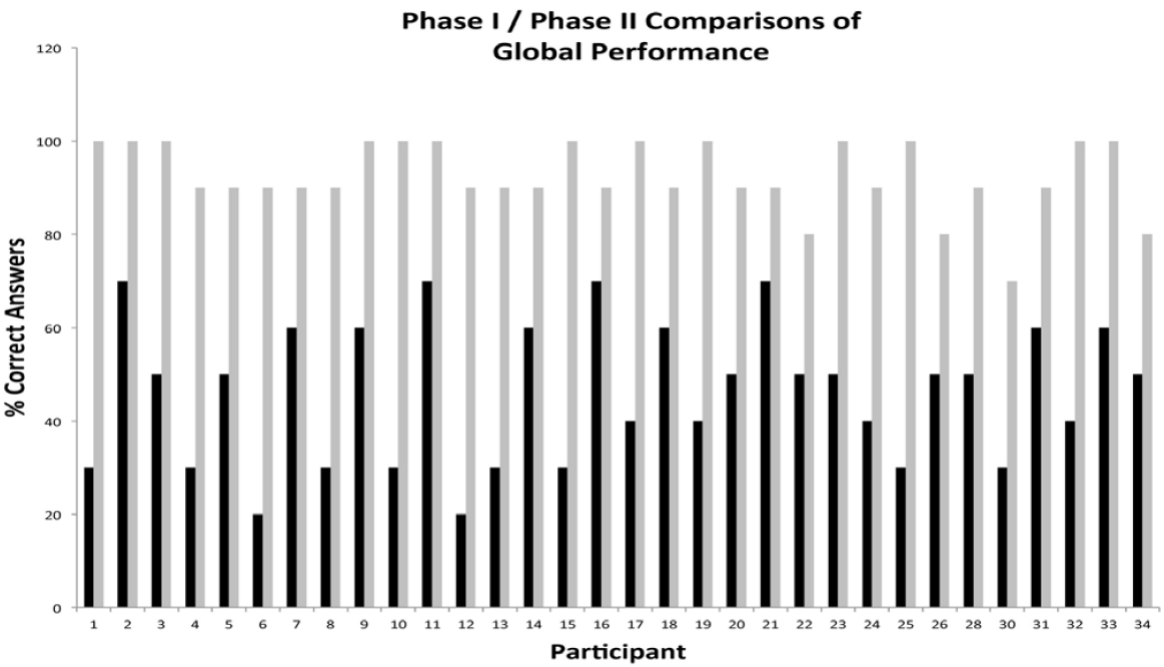
Long Term Knowledge Gains in All Health Areas by Individual Hispanic Participants Nine Months Following Educational Intervention.

Figure 10 compares global knowledge scores upon completion of the first phase with global scores upon completion of instruction 9 months after completion of the initial intervention. An impressive 100% of participants showed improved global performance scores during phase II when compared to phase I of the educational program (Figure 10). The concept areas which showed repeated deficiencies were hidden sources of cholesterol and body mass index, two areas identified as areas of deficiency for the group during pre-testing evaluation.

## Discussion

The present study summarizes the results of a culturally-tailored educational intervention completed in Shelbyville, KY to address the health concerns of Hispanic residents and to improve gaps in health knowledge that contribute to below-basic health literacy. The program was offered to underserved, disadvantaged Hispanic immigrants and focused on risk perceptions of the complex interactions between environment, culture, genetics, and disease. Cultural tailoring entailed four essential elements: 1) Translation of all educational materials into Spanish; 2) Gathering at locations familiar to participants where they felt comfortable and secure; 3) Exclusive communication in Spanish, the language of preference of participants; and 4) Follow up instruction.

The results showed that culturally-tailored health education can effectively improve pre/post testing performance in this cohort. Similar findings have been documented in other studies where pre/post testing designs have been used to evaluate the impact of educational interventions on knowledge acquisition and/or disease outcomes [12-15]. While our study was not limited by excessive drop-out rates or differences in sampling conditions, conventional pre/post testing designs carry inherent limitations. Of most concern are the possibilities that instruction may be targeted “to the test” and that improved performance reflects experiential knowledge or maturation from the exercise of test taking rather than true knowledge of the subject matter. To overcome some of these limitations and to evaluate knowledge retention over time, a second phase of instruction was offered. Importantly, the results of pre-testing performance along with global pre/post testing performance during phase II indicate that participants retained knowledge over time, albeit only documented over a nine month period. As such, culturally-tailored education offered in sequential phases may be a useful strategy for sustained engagement and knowledge retention of disadvantaged Hispanic populations. The need for follow-up instruction and reinforcement is emphasized by the finding that aggregate post-test performance after completion of individual modules was better than global performance upon completion of phase I and global performance after completion of the second phase was consistently high. On the basis of these findings we suggest that specific pathways for successful implementation of health education programs should consider incorporation of one or more of the essential elements identified in the present study.

While this recommendation is supported by the findings, inherent limitations of the study design must be considered. The one-group, pre/post test design applied to a small number of subjects limits assessment of the impact of the intervention on a larger scale. However, data collection in two distinct phases separated in time can be used to reduce the response shift bias inherent to pre/post test designs and to achieve a more accurate assessment of “true” knowledge acquisition. Furthermore, attrition bias is likely not a significant confounder of the study given that 88% of participants re-enrolled in the program. While the study design used was limited in external validity in the absence of external control, the evaluation of pre-test knowledge in two phases afforded some degree of internal validity and controlled for subject selection and variability. Importantly, the lack of standardized health literacy measures for the intervention can be considered a significant weakness. It should be noted, however, that existing methodologies do not necessarily measure health literacy accurately since they mostly function as screening tools applied only once [16]. Lastly, the lack of assessment of changes in related behaviors and/or clinical indicators is a weakness that limits our ability to evaluate in a meaningful way the impact of the intervention on all of the key elements of the health literacy spectrum.

Although health literacy assessment using standardized methodology was not completed, significant deficits in several health literacy indicators were identified in the Shelbyville Health Assessment [11]. The Institute of Medicine (IOM) defines health literacy as “the degree to which individuals have the capacity to obtain, process, and understand basic health information and services needed to make appropriate health decisions” [9]. The IOM report extends the definition to include reading and writing, or “print literacy”; listening and speaking, or “oral literacy”; numeracy; and cultural and conceptual knowledge. The demands for reading, writing, and numeracy skills are intensified due to complexities within the health-care systems, continued advances in scientific discovery, and the introduction of new technologies that have revolutionized the provision of medical care [9]. Health literacy is also strongly contextual, as a person may function adequately at home or work, but be incompetent when faced with complex medical information. Clearly, the American public continues to show significant gaps in health literacy that recognize no racial, ethnic or economic boundaries. Low literacy is linked to unhealthy behaviors, inability to act on prevention and treatment options to manage chronic disease, and promotion of health disparities as they relate to environmental exposures. In our own experience, inadequate health literacy accounts for poor understanding of the medical conditions faced by individuals, poor recall and comprehension of the advice and instructions given during a clinical encounter, persistence of faulty health beliefs that interfere with care, and inability to assimilate information in ways that empower the patient and allow them to manage their health conditions. Noted deficiencies in health literacy are magnified when considering the language, legal and socioeconomic barriers faced by immigrants entering this country, and most importantly, when the complexity of environmental factors and genetics is superimposed upon already disadvantaged populations. It will be important to determine in the future the impacts of the intervention on behaviors and clinical outcomes.

In this study, primary prevention efforts focused on community education with a focus on cardiovascular diseases, diabetes, nutrition, metabolic syndrome and sexually transmitted diseases, topics of interest by the residents. Cardiovascular diseases, cancer and diabetes, along with respiratory diseases, account for 29 million deaths worldwide [17,18]. Of relevance within the context of the Shelbyville program is that the major diseases faced by populations worldwide, are also major causes of morbidity and mortality among Hispanics in this community. The evidence presented shows that translation of scientific knowledge into practical and useful information that is rooted on health prevention and promotion requires strong partnerships between the community and the providers working with them to promote health and assist in managing their medical problems.

Culturally-tailored engagement is an essential element for the development and implementation of strategies to address the health challenges faced by minorities and disadvantaged communities. Recognizing that improvement in health literacy requires collaborative efforts, the important role played by community-based organizations working directly with disadvantaged residents needs to be emphasized. In our case, in-depth discussions with the leadership of these communities helped to identify areas of common interest and to establish bridges that cultivate trust and understanding among all parties. Lastly, it is important to recognize that trust building is an ongoing process that requires continued vigilance and dedication and is an absolute prerequisite for maximization of community involvement leading to improvement of individual and community health.

A major achievement of this intervention was the creation of culturally-tailored educational curricula for dissemination of health knowledge among a disadvantaged Hispanic population in KY. The quantitative data generated from pre/post testing indicated that participants understood and assimilated the information presented both on a short-term basis, as well as several months after completion of the intervention. This suggests that oral and written literacy among participants facilitated the educational intervention. While the Shelbyville program cannot be directly compared to standardized programs given that language is a major barrier in the education of immigrant Hispanics with limited English proficiency, the essential elements identified in our study provide insights into the theory and practice of cultural tailoring to improve health knowledge in efforts to address health literacy deficits and health disparities. As such, the culturally-tailored model of education employed in Shelbyville may be an effective means for dissemination of sustainable, community-wide health prevention for comparable community settings.
